# Case of Ligature of Common Carotid, for Removal of Parotid Gland

**Published:** 1847-09

**Authors:** A. B. Shipman

**Affiliations:** Professor of Surgery in Indiana Medical College


*Case of Ligature of Common Carotid, for removal of Parotid Gland.*—By A. B. Shipman, M. D., Professor of Surgery in Indiana Medical College.—(Communicated by Dr. Norris.)—Mrs. ——, ætat.

70, spare habit, but good general health, had a tumour at the angle of jaw, of four years' standing. (She resided in Tully, Onondaga Co., New York.) The tumour was about the size of an orange, very hard, with lancinating pains through it. Diagnosis, schirrus of the parotid gland. It was determined to extirpate it. Previous to extirpation it was decided to tie the carotid, which was done by myself, and Dr. Narmon Van Dusen, of Tully. At the commencement of the operation, considerable hemorrhage attended, but, the operation was finished, and the patient recovered, the wound healed, and the ligature came away on the 28th day of the operation. The patient was well one year from the operation, but I understood the tumour returned again in the course of two years, and she finally sunk under it. But she recovered perfectly from the operation of tying the carotid. This was in May, 1844, and has never been reported before.—*Ibid.*

